# Mental health, sexual identity, and interpersonal violence: Findings from the Australian longitudinal Women’s health study

**DOI:** 10.1186/s12905-017-0452-5

**Published:** 2017-09-30

**Authors:** Laura A. Szalacha, Tonda L. Hughes, Ruth McNair, Deborah Loxton

**Affiliations:** 1 Office of Nursing Research, College of Nursing, University of Arizona, Tucson, USA; 2 Nursing and Psychiatry, School of Nursing, Columbia University, New York City, USA; 3 Global Health Research, School of Nursing, Columbia University, New York City, USA; 4 Department of General Practice, The University of Melbourne, Melbourne, Australia; 5 Australian Longitudinal Study on Women’s Health, University of Newcastle, Melbourne, Australia; 6 Research Centre for Generational Health and Ageing, University of Newcastle, Callaghan, Australia

**Keywords:** Interpersonal violence, Female sexual identity, Stress, Depression, Australian longitudinal Women’s health study

## Abstract

**Background:**

We examined the relationships among experiences of interpersonal violence, mental health, and sexual identity in a national sample of young adult women in Australia.

**Methods:**

We used existing data from the third (2003) wave of young adult women (aged 25–30) in the Australian Longitudinal Study on Women’s Health (ALSWH). We conducted bivariate analyses and fit multiple and logistic regression models to test experiences of six types of interpersonal violence (physical abuse, severe physical abuse, emotional abuse, sexual abuse, harassment, and being in a violent relationship), and the number of types of violence experienced, as predictors of mental health. We compared types and number of types of violence across sexual identity subgroups.

**Results:**

Experiences of interpersonal violence varied significantly by sexual identity. Controlling for demographic characteristics, compared to exclusively heterosexual women, mainly heterosexual and bisexual women were significantly more likely to report physical, sexual, and emotional abuse. Mainly heterosexual and lesbian women were more likely to report severe physical abuse. Mainly heterosexual women were more than three times as likely to have been in a violent relationship in the past three years, and all three sexual minority subgroups were two to three times as likely to have experienced harassment. Bisexual women reported significantly higher levels of depression than any of the other sexual identity groups and scored lower on mental health than did exclusively heterosexual women. In linear regression models, interpersonal violence strongly predicted poorer mental health for lesbian and bisexual women. Notably, mental health indicators were similar for exclusively heterosexual and sexual minority women who did not report interpersonal violence. Experiencing multiple types of interpersonal violence was the strongest predictor of stress, anxiety and depression.

**Conclusions:**

Interpersonal violence is a key contributor to mental health disparities, especially among women who identify as mainly heterosexual or bisexual. More research is needed that examines within-group differences to determine which subgroups are at greatest risk for various types of interpersonal violence. Such information is critical to the development of effective prevention and intervention strategies.

## Background

Violence against women is one of the least visible but most widespread forms of violence in the world. The World Health Organization has noted global epidemic rates of violence against women and highlighted the serious, long-term effects of such violence by positioning it as a leading worldwide public health issue [1]. The Australian component of the International Violence Against Women Survey found that 48% of the female population had experienced at least one incident of physical abuse in their lifetime, and 34% had experienced at least one incident of sexual violence [2]. In Victoria, Australia, intimate partner violence is the leading contributor to death, disability, and illness in women aged 15 to 44 years [3].

The relationship between violence and poor mental health is well established in the literature [4–11]. For example, in analyses of the mid-aged women in the Australian Longitudinal Study on Women’s Health (ALSWH), Loxton, Schofield, and Hussain found strong and consistent relationships among domestic violence, poorer mental health, and depression [12]. In addition, results of a recent meta-analysis conducted by MacIsaac and colleagues showed a strong association between interpersonal violence and suicide among women [13]. Compared to their heterosexual counterparts, sexual minority women are at increased risk for mental health problems such as depression, anxiety disorders, and substance abuse [14–17]. One potential explanation for mental health disparities among sexual minority women is elevated rates of interpersonal violence.

In the past decade, evidence has accumulated indicating that a disproportionately high number of sexual minority women (those who do not identify as exclusively heterosexual) are victims of physical and sexual abuse [10, 11, 14, 18–20]. Although the factors that account for this disparity are poorly understood, researchers have found strong associations between violence and substance abuse, depression, and anxiety among sexual minority women [10, 11, 15, 21–26].

Studies conducted in the United States that compared experiences of violence in sexual minority and heterosexual women have found compelling evidence of sexual-orientation-related disparities. In one such study, Balsam, Rothblum, and Beauchaine found that lesbian, gay, and bisexual participants were more likely than their heterosexual siblings to report childhood physical abuse (CPA), childhood sexual abuse (CSA), ever being assaulted by a partner, and a history of sexual coercion or rape [19]. Similarly, Moracco found that a combined sample of lesbian and bisexual women reported significantly higher rates of physical and sexual assault by a stranger or known person than did heterosexual women [27]. In addition to the growing consensus that sexual minority women are more likely than heterosexual women to experience violence, results of recent studies suggest that rates vary, sometimes substantially, across sexual minority subgroups.

With few exceptions, most research on violence among SMW has not assessed subgroup differences [10, 11, 22, 28]. Studies that examine within group differences have most often focused on comparisons between lesbian and bisexual women. For example, Balsam and colleagues compared data from bisexual and lesbian women and found that bisexuals were significantly more likely than lesbians to report sexual coercion and sexual assault in adulthood [19]. Similarly, Hughes and colleagues found that bisexual women reported significantly higher rates of CSA, CPA, partner violence, and non-partner violence than did heterosexual women, whereas only CSA differed between lesbian and heterosexual women [22]. A focus on binary classifications of sexual identity (e.g., sexual minority vs. heterosexual, or lesbian vs. bisexual) may mask important variation across subgroups of sexual minority women. In addition, recent research has begun to document health disparities among women who identify as mainly or mostly heterosexual. For example, using data from a U.S. community-based sample, Austin and colleagues found that young women, aged 18 to 24 years, who identified as “mostly heterosexual” reported significantly higher rates of CSA than did those who identified as heterosexual [23]. Although not a study of violence, Hughes, Szalacha, and McNair found that sexual minority women in Australia who identified as mainly heterosexual were significantly more likely than exclusively heterosexual women to report at-risk drinking (≥ 15 drinks per week) and binge drinking [16]. Similarly, Hughes, Wilsnack and Kristjanson found that compared with their exclusively heterosexual counterparts who identified as mostly heterosexual were 2–4 times as likely to report every substance use outcome assessed except lifetime treatment for an alcohol-related problem [29].

In summary, most research has not assessed subgroup differences within sexual minority populations. In addition, very few studies have examined multiple types of violence or multiple mental health outcomes across sexual minority subgroups.

Therefore, using data from the Australian Longitudinal Study on Women’s Health (ALSWH) we addressed the following research questions:

1. Do experiences of interpersonal violence (physical abuse, severe physical abuse, emotional abuse, sexual abuse, harassment, being in a violent relationship, and number of types of violence experienced (individual types of violence and the number of different types) vary significantly across sexual identity subgroups?; and,
2. To what extent is the association between sexual identity and poorer mental health accounted for by experiences of interpersonal violence?

Examining differences between heterosexual and sexual minority women can improve understanding of sexual identity-related health disparities. In addition, understanding sexual minority subgroup differences can inform the development of targeted prevention and intervention strategies.

## Methods

The ALSWH is a prospective study that began in 1996 with the goal of tracking the health of women in three age cohorts (18–23, 45–50, and 70–75 at baseline) for at least 20 years [30]. The study samples are drawn from the database of Medicare Australia, the universal provider of basic health insurance, which involves all people in Australia (including all citizens and permanent residents). Sampling from the population was random within each age group, with oversampling from rural and remote areas to allow for statistical comparisons of the circumstances and health of city and country participants. Mailed surveys are completed approximately every three years. Further details of the study design and methods have been reported elsewhere [30–32]. The current analyses focus on data from the third survey of the young cohort because it was the most recent survey in which sexual identity was assessed. A total of 9074 participants completed Survey 3, an estimated response rate of 64%. The young women (aged 25–30) who answered the sexual identity question (*n* = 8850) serve as the analytic sample for this study.

### Measures

#### Sexual identity

Participants were asked which of five categories best described their sexual identity: exclusively heterosexual, mainly heterosexual, bisexual, mainly lesbian, or exclusively lesbian. For the current analyses, participants who identified as mainly lesbian or exclusively lesbian were combined because of the small number of women in the mainly lesbian and exclusively lesbian groups and because of the similarity between the two groups relative to demographic characteristics and the study variables.

#### Experiences of interpersonal violence

The interpersonal violence questions were based on a previously existing battery of questions [33]. Participants were asked whether or not in the previous three years they had experienced the following: (1) physical abuse (e.g., pushed, grabbed, kicked, hit, shoved, slapped, shaken, restrained); (2) severe physical abuse (e.g., beaten up, thrown, choked, burnt, attacked with a fist, knife or gun); (3) emotional abuse (e.g., called names, threatened with harm or death, humiliated, bullied, criticized, locked up/isolated, refused access to work, medical care or money, told that their children or pets would be harmed); (4) sexual abuse (e.g., rape or attempted rape, sexual assault, forced to engage in unwanted sexual practices); and (5) harassment (e.g., stalking, loitering, interfering with property, offensive mail or telephone calls). An additional question asked whether participants “had *ever* been in a violent relationship with a partner or spouse” (violent relationship was not defined for survey participants). Responses to the six items were summed to create a continuous measure of interpersonal violence experiences (range 0–6).

### Mental health outcomes

#### Stress

The Perceived Stress Questionnaire for Younger Women was developed specifically for the ALSWH. Studies of the younger cohort conducted by Bell and Lee [34, 35] have demonstrated that the scale is valid and reliable (e.g., Cronbach’s alpha = 0.74). Respondents were asked 11 questions about how stressed they have felt over the previous 12 months in relationship to several life domains, such as family of origin, relationships with partner/spouse and others, health, work/employment, study, and motherhood/children. Responses were on a 5-point Likert-type scale ranging from 0 = *not at all stressed (or not applicable)* to 4 = *extremely stressed*, with higher scores indicating higher levels of stress.

#### Anxiety

Participants were asked a single question about how often in the previous 12 months they had experienced episodes of intense anxiety (anxiety symptoms; 0 = *never* to 3 = *often*).

#### Depression

A 10-item version of the Center for Epidemiologic Studies Depression Scale (CES-D) was used to assess current (past 4 weeks) depression. The CES-D was developed specifically to identify depressive symptoms and screen for depression in non-clinical populations. Scale scores range from 0 to 30. The 10-item version of the CES-D used in the ALSWH demonstrated good internal consistency (Cronbach’s alpha = 0.80) and validity [36]. Responses ranged 0–10, with higher scores indicating higher levels of depressive symptoms.

#### Mental health index

To assess overall mental health, we used responses to a 5-item subscale of the well-validated Medical Outcomes 36-item Short Form Survey (SF-36) [37]. Participants were asked to “give the one answer that comes closest to the way you’ve been feeling during the past four weeks.” Response options were on a 6-point Likert-type scale ranging from *none of the time* to *all of the time*. Summed scores ranged from 0 to 100, with higher scores indicating better overall mental health status (Cronbach’s alpha = 0.88).

#### Life satisfaction

Respondents were asked about their satisfaction with seven areas of their life. “In general, are you satisfied with what you have achieved in your life so far” in the areas of work, career, study, family relationships, partner/closest personal relationship, friendships, and social activities. Response options were on a 4-point Likert-type scale ranging from *very dissatisfied* to *very satisfied*, with higher scores indicating greater life satisfaction.

Copies of all of the ALSWH surveys can be found at the study website [38].

### Control variables

Multivariate analyses controlled for the potential confounding effects of education, income, and region of residence. Given the narrow age range of the young cohort sample (25–30 years), age was not included as a control variable. All of the control variables were treated as categorical: education (1 = year 10 or less, 2 = year 12 or equivalent, 3 = Trade/Diploma, 4 = undergraduate degree, and 5 = higher university degree), annual income in Australian dollars (1 = $0 to $15,999, 2 = $16,000–$36,999, 3 = $37,000–$51,999, 4 = $52,000 or more), relationship status (1 = married; 2 = living in a de facto relationship; 3 = separated, divorced, or widowed; or 4 = single), parental status (0 = no child, 1 = one or more children), and region of residence (1 = urban, 0 = rural/remote).

## Data analysis

We compared socio-demographic characteristics, types of interpersonal violence, and mental health outcomes across the four sexual identity groups, using contingency table analyses for the categorical variables and ANOVA and ANCOVA for the continuous variables. We fit six logistic regression models to the data to estimate the relative risk of each type of interpersonal violence based on sexual identity, controlling for residence, education, and income. We fit five linear regression models to predict mental health outcomes by sexual identity and number of types of interpersonal violence, controlling for the same demographic variables. In analyses not including residence as a predictor, the data were weighted by region. Exclusively heterosexual women served as the reference group in all comparisons of sexual identity. We report standardized beta coefficients to facilitate comparison of effect size. Data were analyzed using Stata 10 [39] with an alpha of 0.05 for all statistical tests.

## Results

### Sample socio-demographic characteristics

The majority of participants in the sample identified as exclusively heterosexual, 91.3% (*n* = 8083); 6.4% identified as mainly heterosexual (*n* = 568), 1.1% identified as bisexual (*n* = 100), and 1.1% identified as mainly lesbian or exclusively lesbian (*n* = 99). The distributions of the socio-demographic characteristics differed significantly by sexual identity (see Table 1). Women who identified as exclusively heterosexual were proportionally more likely than those in other groups to be married (44.0%),^1^ to live in rural/remote areas (40.4%), to have children (32.8%), and to report annual incomes of $52,000 or more (40.4%). Lesbian-identified women were significantly older (*M* = 27.5, *SD* = 1.4) than each of the other three sexual identity groups (F_(3, 7605)_ = 2.98, *p* = .03), and were proportionally more likely to have a university or graduate degree (51.5%).

**Table 1:** ALSWH young cohort socio-demographic characteristics, violence experiences and mental health by sexual identity

	Exclusively Heterosexual	Mainly Heterosexual	Bisexual	Lesbian	χ2 Test Statistic	Cramer’s V
	(*n* = 8083)	(*n* = 568)	(*n* = 100)	(*n* = 99)		
	%	%	%	%		
Education						
Year 10 or less	9.9	10.3	20.6	5.2		
Year 12 or equivalent	19.4	19.4	19.6	17.5		
Trade/Diploma	26.7	24.8	26.8	25.8		
University degree	34.2	34.6	25.8	44.3	(*df* = 12)	.031*
Graduate degree	10.7	10.9	7.2	7.2	χ2 = 21.0**	
Income (AUD)						
15,999 or less	1.9	2.4	6.8	4.3		
16,000–36,999	4.8	6.2	13.6	8.6		
37,000–51,999	11.5	12.1	13.6	12.9	(*df* = 9)	.036*
52,000 or greater	81.4	79.4	66.1	74.3	χ2 = 25.7**	
Relationship Status						
Single	32.6	50.0	58.0	62.6		
Married	44.0	18.9	13.0	2.0		
De facto	19.9	25.4	20.0	32.3	(*df* = 9)	.098***
Separated/Divorced	3.5	5.7	9.0	3.0	χ2 = 255.1***	
Parental Status						
No children	67.2	73.8	72.0	93.9	(*df* = 3)	.069***
1 or more children	32.8	26.2	28.0	6.1	χ2 = 43.2***	
Residence						
Urban	59.6	69.7	69.8	69.8	(*df* = 3)	.046***
Rural/Remote	40.4	30.3	30.2	30.2	χ2 = 29.6***	
Violence					(*df* = 9)	
Physical abuse	11.50	24.10	29.00	22.20	χ2 = 110.0***	.111***
Severe physical abuse	2.10	6.50	11.00	10.10	χ2 = 91.3***	.102***
Emotional abuse	20.60	32.40	46.00	28.30	χ2 = 80.8***	.096***
Sexual abuse	2.10	6.20	14.00	3.00	χ2 = 93.5***	.103***
Harassment	9.70	19.50	25.00	21.20	χ2 = 88.2***	.100***
Violent relationship	10.00	24.60	24.50	14.70	χ2 = 130.4***	.123***
	M (SE)	M (SE)	M (SE)	M (SE)	F-test	
Overall number of violent experiences	.44 (.01)^a^	.79 (.04)	1.20 (.09)^b^	.89 (.09)	F_(4,7610)_ = 53.48***	
Mental Health						
Perceived Stress	*.88* .50^a^	1.10 .62	1.34 .69	1.04 .54	F_(3,7587)_ = 53.0**	
Anxiety Symptoms	*1.3* .68^b^	1.5 .86	1.7 .99	1.5 .87	F_(3,7587)_ = 23.9***	
CES-D	6.7 5.1	7.7 5.9	*9.5* 5.9^c^	7.7 5.9	F_(3,7487)_ = 14.9***	
Mental Health Index	71.0 16.7	66.5 18.9	*64.1* 18.4^d^	67.1 18.8	F_(3,7585)_ = 17.5***	
Life Satisfaction	*3.3* .42^e^	3.1 .48	3.0 .51	3.2 .42	F_(3,7585)_ = 33.6***	

^a^Exclusively heterosexual significantly lower than all other groups (η = .021). ^b^Exclusively heterosexual significantly lower thanall other groups (η = .014). ^c^Bisexual significantly higher than all other groups (η = .013). ^d^Bisexual significantly lower than exclusively heterosexual (η = .013). ^e^Exclusively heterosexual significantly higher than mainly heterosexual and bisexual (η = .013)

*p < .05. **p < .01. ***p < .001

### Experiences of interpersonal violence

Both the individual types and the total number of types of interpersonal violence differed significantly by sexual identity. Women in each of the three sexual minority groups were more likely than exclusively heterosexual women to report every type of interpersonal violence (physical abuse, severe physical abuse, emotional abuse, harassment, sexual abuse, and partner violence; see Table 1). Bisexual and mainly heterosexual women were most likely to report experiencing each of the six types of interpersonal violence. Substantially fewer exclusively heterosexual women reported physical abuse (11.5%) than did mainly heterosexual (24%), bisexual (29%), or lesbian (22%) participants. Similarly, between 6.5% and 11% of the sexual minority groups reported *severe* physical abuse, compared with only 2.1% of exclusively heterosexual women. Bisexual participants were significantly more likely than the other three sexual identity groups to report both emotional abuse and sexual abuse. Harassment was reported by 9.7% of exclusively heterosexual, 19.5% mainly heterosexual, 25.0% bisexual, and 21.2% of lesbian participants. Similarly, 10.0% of exclusively heterosexual participants reported ever having been in a violent relationship, compared with 24.6% mainly heterosexual, 24.5% bisexual, and 14.7% lesbian participants. The number of types of interpersonal violence reported also differed by sexual identity; exclusively heterosexual women reported significantly fewer experiences (*M* = .44, *SE* = .01) and bisexual women reported significantly more experiences (*M* = 1.20, *SE* = .09) than each of the other groups (η = .02) (F_(4,8885)_ = 53.48, *p* < .001).

We fit six logistic regression models to the data to estimate the relative risk of each type interpersonal violence based on sexual identity. Results, summarized in Table 2, indicate a greater burden of interpersonal violence—in the main, two to three times greater—in the lives of sexual minority women. This was true for physical abuse (adjusted odds ratios [AOR; odds ratios adjusted for other variables in the model] ranged from 2.14 to 2.51), harassment (AORs ranged from 2.35 to 3.30), and violent relationships (AORs ranged from 1.93 to 3.07). In comparisons of lesbian and exclusively heterosexual women, the only non-significant differences were for emotional abuse and sexual abuse. Conversely, the largest differences were in comparisons of exclusively heterosexual and lesbian women on severe physical abuse (AOR = 6.40) and exclusively heterosexual and bisexual women on sexual abuse (AOR = 5.72). Given the very wide 95% confidence intervals, these results should be interpreted somewhat cautiously.

**Table 2:** Experiences of violence reported by sexual identity, controlling for socio-demographic characteristics^a^

	Physical violence AOR (95% CI)	Severe physical violence AOR (95% CI)	Emotional abuse AOR (95% CI)	Sexual abuse AOR (95% CI)	Harassment AOR (95% CI)	Violent relationship AOR (95% CI)
Sexual Identity						
Exclusively Heterosexual	Referent	Referent	Referent	Referent	Referent	Referent
Mainly Heterosexual	*2.51 (2.00–3.15)*	*3.59 (2.37–5.42)*	*1.81 (1.47–2.21)*	*3.32 (2.19–5.02)*	*2.35 (1.85–3.00)*	*3.07 (2.43–3.89)*
Bisexual	*2.41 (1.41–4.12)*	*4.74 (2.11–10.64)*	*3.10 (1.96–4.90)*	*5.72 (2.68–12.19)*	*3.30 (1.95–5.60)*	*2.33 (1.32–4.13)*
Lesbian	*2.14 (1.25–3.66)*	*6.40 (2.99–13.67)*	1.47 (.90–2.41)	1.73 (.41–1.42)	*2.60 (1.51–4.45)*	*1.93 (1.03–3.61)*

^a^Education, income and residence

### Mental health and sexual identity

Mental health indicators also varied significantly across the sexual identity subgroups. For each of the five indicators (stress, anxiety symptoms, depression, overall mental health, and life satisfaction), mainly heterosexual, bisexual, and lesbian participants scored significantly poorer than did exclusively heterosexual participants. Among the three sexual minority groups, bisexual participants reported the highest levels of stress (*M* = 1.3, *SD* = .69, (F_(3,7587)_ = 53.0, *p* < .001)), anxiety symptoms (*M* = 1.7, *SD* = .99, F_(3,7587)_ = 23.9, *p* < .001) and depression (*M* = 9.5, *SD* = 5.9, F_(3,7487)_ = 14.9, *p* < .001), and the lowest scores on the mental health index (*M* = 64.1, *SD* = 18.4, (F_(3,7585)_ = 17.5, *p* < .001)) and life satisfaction scale (*M* = 3.0, *SD* = .51, (F_(3,7585)_ = 33.6, *p* < .001).

### Interpersonal violence and mental health

Experiences of interpersonal violence significantly predicted poorer mental health. Women who had experienced any of the six types of interpersonal violence scored significantly higher on stress, anxiety symptoms and depression, and significantly lower on the mental health index and life satisfaction scales. There were moderate positive correlations between the number of types of interpersonal violence and stress (*r* = .33, *p* < .001), anxiety symptoms (*r* = .21, *p* < .001), and depression (*r* = .29, *p* < .001), and moderate negative correlations between the number of types of interpersonal violence and the mental health index (*r* = −.26, *p* < .001) and life satisfaction scale (*r* = −.28, *p* < .001) scores.

### Interpersonal violence, mental health, and sexual identity

Relationships between the individual types of interpersonal violence experiences, and the number of types of interpersonal violence and poorer mental health varied across sexual identity groups. We fit five linear multiple regression models to the data to systematically examine the relationships among each type of interpersonal violence, sexual identity and mental health (see Table 3).

**Table 3:** Mental health associations with types of violence and sexual identity, and number of violent experiences and sexual identity, controlling for education, income, and residence in multiple regression models

	Perceived	Perceived	Anxiety	Anxiety			Mental health	Mental health	Life	Life
	Stress	Stress	Symptoms	Symptoms	CES-D	CES-D	Index	Index	Satisfaction	Satisfaction
	M1	M2	M3	M4	M5	M6	M7	M8	M9	M10
	Std β	Std β	Std β	Std β	Std β	Std β	Std β	Std β	Std β	Std β
Type of Violence										
Physical abuse	.08***		*0.02*		.07***		−.06***		−.05***	
Severe physical abuse	0.02		0.02		.03*		−.03*		−0.01	
Emotional violence	.22***		.13***		.15***		−.17***		−.16***	
Sexual violence	.03**		.04***		0.02		−0.02		−.04**	
Harassment	.08***		.07***		.07***		−.05***		−.05***	
In a violent relationship	.06***		.05***		.03*		−.03*		−0.02	
Number of Violent Experiences		.324***		.194***		.251***		−.240***		−.223***
Sexual Identity										
Exclusively heterosexual	Referent	Referent	Referent	Referent	Referent	Referent	Referent	Referent	Referent	Referent
Mainly heterosexual	.06***	.06***	.06***	.06***	.03*	.03*	−.05***	−.05***	.06***	.06***
Bisexual	.06***	.06***	.04**	.04***	0.02	0.02	−0.02	−0.02	−.03*	−.03*
Lesbian	.01	.01	.02	.01	.01	.01	−.01	−.01	−.01	−.01

*p < .05. **p < .01. ***p < .001

Almost every type of interpersonal violence significantly predicted each of the indicators of mental health. In particular, emotional violence was a strong predictor, with estimated standardized coefficients ranging from Std β = .13, (*t* = 11.1, *p* < .001) to Std β = .22, (*t* = 16.6, *p* < .001). The weakest predictor was severe physical abuse, which significantly predicted higher depression and mental health index scores, with estimated standardized coefficients of Std β = .03, (*t* = 2.53, *p* < .001) and Std β = .03, (*t* = 2.23, *p* < .001) respectively. Women who identified as mainly heterosexual scored significantly higher on stress, anxiety and depression and significantly lower on the mental health index and the life satisfaction scale. Bisexual identity significantly predicted higher stress and anxiety, and lower life satisfaction scores. Lesbian identity was not a significant predictor of difference in any mental health measure after controlling for level of interpersonal violence.

We fit five additional linear multiple regression models to the data, examining the relationships among the number of types of interpersonal violence experiences, sexual identity, and mental health (also presented in Table 3). The number of types of interpersonal violence was the single strongest predictor of perceived stress (Std β = .32, *t* = 22.5, *p* < .001), anxiety symptoms (Std β = .19, *t* = 12.4, *p* < .001), depression (Std β = .25, *t* = 17.5, *p* < .001), poorer overall mental health (Std β = −.24, *t* = 16.1, *p* < .001), and life satisfaction (Std β = −.22, *t* = 13.6, *p* < .001). Notably, many of the sexual identity differences in the mental health outcomes found in bivariate analyses were no longer significant once the number of types of interpersonal violence was included in the models. Identifying as lesbian was not a significant predictor of any mental health outcome. Bisexual identity was significantly associated with three mental health outcomes: perceived stress (Std β = .06, *t* = 5.27, *p* < .01), anxiety symptoms (Std β = .04, *t* = 3.27, *p* < .001), and life satisfaction (Std β = −.22, *t* = 2.34, *p* < .05). Of particular note is the fact that mainly heterosexual self-identity remained a significant predictor of every mental health outcome. Specifically, mainly heterosexual identity predicted higher perceived stress (Std β = .06, *t* = 5.39, *p* < .001), greater anxiety symptoms (Std β = .06 *t* = 4.97, *p* < .001), higher level of depression (Std β = .03, *t* = 2.28, *p* < .05), poorer overall mental health (Std β = −.05, *t* = 3.80, *p* < .001), and lower life satisfaction (Std β = −.06, *t* = 5.27, *p* < .001).

## Discussion

These findings from a population-based national sample contribute to the growing body of evidence supporting the disproportionate burden of interpersonal violence on sexual minority women [10, 14, 16, 27, 40]. Such findings are consistent with minority stress theory which attributes sexual-orientation-related mental health disparities to societal and contextual factors [41]. Further, variations in prevalence of interpersonal violence across sexual minority subgroups point to the need for additional research aimed at greater understanding of within-group differences.

Results of the study support our first hypothesis that, relative to exclusively heterosexual women, sexual minority women (mainly heterosexual, bisexual, and lesbian) would be at heightened risk of interpersonal violence, and that experiences of violence would differ across sexual minority subgroups. Indeed, sexual minority women in the study were more likely to report every type of interpersonal violence assessed in the study. Some of this difference may be attributed to the increased vulnerability to interpersonal violence that comes with visibility as a sexual minority person [40, 42, 43]. Researchers have found that sexual minority individuals who are more open about their sexual orientation (i.e., more visible) are more likely to experience victimization [24, 44]. Lesbian women are more likely than bisexual, and presumably more likely than mainly heterosexual women, to disclose their sexual identity—a factor that may explain, at least in part, lesbians’ higher rates of some types of interpersonal violence [45]. However, this does not explain higher rates of some types of interpersonal violence amongst bisexual and mainly heterosexual women, whose sexual orientation is less publicly visible.

Although we were unable to ascertain the sex of perpetrators of partner violence, it is reasonable to speculate that bisexual and mainly heterosexual women are more likely than lesbian women to have been in recent (past three years) violent relationships with male partners. Findings in the literature, however, suggest that partner violence may occur at the same rate in opposite- and same-sex couples; or, in same-sex male couples, at higher rates [19, 46, 47]. Therefore, factors in addition to partner sex likely influence risk of interpersonal violence among sexual minority women. Because mainly heterosexual and bisexual women are on the whole less likely to be affiliated with lesbian communities and may also feel alienated from the heterosexual community, they may experience heightened feelings of isolation and loneliness that contribute to risk of IPV and to negative mental health outcomes [29, 48]. In addition, as discussed in the background section, mainly heterosexual women are at heightened risk of alcohol and other drug use, which may render them more vulnerable to violence. Additional research examining sexual identity subgroup differences in health behaviors and health outcomes is needed to more fully understand these relationships.

In regard to hypothesis 2 that experiences of violence would predict poorer mental health we found that the number of types of violence was the strongest predictor of mental health and that this association held even when controlling for demographic characteristics and sexual identity. These findings suggest that interpersonal violence is a robust predictor of poor mental health, regardless of sexual identity. In addition, the absence of a significant interaction effect of sexual identity on the relationship between interpersonal violence and mental health suggests that the effects of interpersonal violence do not differ substantially by sexual identity. Interpersonal violence contributes to poor mental health among all women.

As a caveat to the findings indicating a strong relationship between interpersonal violence and poor mental health, we found that even after controlling for interpersonal violence, mainly heterosexual women reported higher levels of stress, anxiety, and depression, and lower overall mental health and life satisfaction. The standardized coefficients for the effect of mainly heterosexual identity ranged from .03 to .06, whereas the standardized coefficients for the effect of interpersonal violence ranged from .19 to .32. This was also true, to a lesser degree, for bisexual women who, after controlling for interpersonal violence in the models, reported higher levels of stress and anxiety, and lower life satisfaction. These findings suggest that there exists a complex array of influences on sexual minority women’s mental health, including marginalization and lower levels of social connectedness, which are likely more pronounced among bisexual and mainly heterosexual women than among lesbian women.

Our findings are consistent with a those from a growing number of studies showing that women who identify as mainly heterosexual are at increased risk of poor mental health [10, 16, 49, 50]. In previous analyses of young women in the ALSWH, mainly heterosexual women were more likely than exclusively heterosexual and lesbian women to report self-harm and feeling that life was not worth living [16]. Results of those analyses also indicated that mainly heterosexual women had lower levels of social support than did exclusively heterosexual or lesbian women. Similarly, Corliss and colleagues found that mostly heterosexual women were less likely to report social support from friends and family and more likely to report a history of and treatment for depression [49]. The lower levels of social support found in these studies lends evidence to the supposition that mainly heterosexual women are even more likely than other sexual minority groups to be marginalized and isolated, and that these experiences likely contribute to heightened risk of adverse health outcomes.

### Strengths and limitations

This study has a number of important strengths. The sample was recruited without explicit sexual orientation criteria. The data are from a national sample, rather than being defined by a region or occupational group, and thus includes women that represent a wide range of geographic, socioeconomic, and personal circumstances. The large sample size afforded the opportunity to examine differences across sexual minority subgroups. Sexual minority subgroup sample sizes, to our knowledge, are among the largest of any national sample. (The National Epidemiologic Survey on Alcohol and Related Conditions [NESARC], conducted in the United States, has comparable numbers of lesbian and bisexual women.) In addition, whereas most studies, particularly most national studies (including the NESARC), assess sexual identity based on a three-category response option (lesbian, bisexual, heterosexual), the question used in the current study allowed women to choose from a wider range of sexual identity response options. Our findings, as well as those from other studies indicating heightened risk among mostly heterosexual women, point to the importance of including this group in studies aimed at understanding health disparities among sexual minorities [10, 16, 22, 37, 49, 51].

Finally, most of the measures were well established scales (e.g., CES-D) or have been validated in previous waves of the ALSWH.

Despite these strengths, several limitations should be considered when interpreting the findings. First, the response rate of the original survey of the young cohort was low (40%–41% for the baseline and 68% and 64% of the baseline at the second and third follow-up surveys). Additionally, 2.4% (*n* = 211) of participants did not respond to the question regarding their sexual orientation (i.e., they indicated that they were unsure of their sexual identity or they refused to answer the question). Each of these factors could limit the generalizability of the findings. It is important to note, however, that Powers and Loxton’s examination of the impact of attrition in the ALSWH concluded that the biases were insufficient to preclude meaningful longitudinal analyses [52]. Second, although sexual identity was assessed, other major sexual orientation dimensions, such as sexual attraction and behavior, were not. Having information about sexual attraction and sexual behavior would have permitted more nuanced assessments of sexual orientation-related mental health risks and could have been used to better understand how women in the study self-identified—information that would have been particularly helpful in understanding the women who identified as mainly heterosexual.

The current study was conducted using cross sectional data, so no inferences as to causation can be made. For example, although it is possible that emotional abuse leads to poor mental health outcomes, it is also possible that poor mental health might lead to heightened perceptions or reporting of emotional abuse.

Harassment experiences are likely related, at least in part, to sexual orientation discrimination; however, given the wording of the question, we were unable to distinguish the perceived causes of harassment. Similarly, the study is limited by the lack of information regarding the gender of perpetrators of interpersonal violence.

Finally, there are likely other important variables (e.g., heavy drinking or drug use) not included in the analyses that are associated with both victimization and mental health.

## Conclusions

Given that sexual minority women are at higher risk of interpersonal violence and poor mental health outcomes, violence prevention and mental health services targeting women with diverse sexual identities are crucial. Our findings highlight sexual-orientation-related disparities in experiences of interpersonal violence and point to important subgroup differences that go beyond a binary categorization of sexual orientation. In addition, study findings lend evidence to the assertion that social and contextual factors such as interpersonal violence, rather than sexual minority status, accounts for some of the mental health disparities observed among sexual minority women. The growing body of findings indicating heightened risk of adverse mental health outcomes among mainly heterosexual women suggests that this subgroup may have additional risk factors not assessed in this study. The wellbeing of women who identify as mainly heterosexual warrants further research to better understand women who choose this identity category and the factors that contribute to their heightened risk of violence and poor mental health. Although the health impact of interpersonal violence on women in the general population is increasingly acknowledged and taken into account in clinical practice, mental health referral pathways for sexual minority women are less clear. In particular, research has found that clinicians tend to assume that same-sex partnered women are at low or no risk of intimate partner violence [53]. To more fully understand sexual-orientation-related mental health disparities, additional research is needed that addresses risk behaviors, relationship characteristics, and other psychosocial factors associated with women’s diverse sexual identities.

## Abbreviations

ALSWH: Australian Longitudinal Study on Women’s Health
CES-D: Center for Epidemiologic Studies Depression Scale
CPA: Childhood physical abuse
CSA: Childhood sexual abuse
LGB: Lesbian, Gay and Bisexual
NESARC: The National Epidemiologic Survey on Alcohol and Related Conditions
SF-36: Medical Outcomes 36-item Short Form Survey

## Footnotes

1 Same-sex couples are prevented from marrying in Australia. Same-sex unions are treated as de facto unions under the Australian federal law, though each Australian state and territory is entitled to create their own laws with respect to same-sex relationship registers and same-sex partnership schemes.
